# Tumour biomarkers: association with heart failure outcomes

**DOI:** 10.1111/joim.13053

**Published:** 2020-05-05

**Authors:** C. Shi, H. H. van der Wal, H. H. W. Silljé, M. M. Dokter, F. van den Berg, L. Huizinga, M. Vriesema, J. Post, S. D. Anker, J. G. Cleland, L. L. Ng, N. J. Samani, K. Dickstein, F. Zannad, C. C. Lang, P. L. van Haelst, J. A. Gietema, M. Metra, P. Ameri, M. Canepa, D. J. van Veldhuisen, A. A. Voors, R. A. de Boer

**Affiliations:** ^1^ Department of Cardiology Uni University Medical Center Groningen Groningen the Netherlands; ^2^ University of Groningen University Medical Center Groningen Groningen the Netherlands; ^3^ Department of Cardiology Berlin‐Brandenburg Center for Regenerative Therapies German Centre for Cardiovascular Research (DZHK) Partner site Berlin Charité Universitätsmedizin Berlin Berlin Germany; ^4^ National Heart & Lung Institute Royal Brompton & Harefield Hospitals Imperial College London UK; ^5^ Robertson Institute of Biostatistics and Clinical Trials Unit University of Glasgow Glasgow UK; ^6^ Department of Cardiovascular Sciences University of Leicester Leicester UK; ^7^ NIHR Leicester Biomedical Research Centre Glenfield Hospital Leicester UK; ^8^ University of Bergen Stavanger University Hospital Stavanger Norway; ^9^ Clinical Investigation Center 1433 French Clinical Research Infrastructure Network Investigation Network Initiative‐Cardiovascular and Renal Clinical Trialists Centre Hospitalier Regional et Universitaire de Nancy Vandoeuvre les Nancy France; ^10^ Division of Molecular and Clinical Medicine Ninewells Hospital and Medical School University of Dundee Dundee UK; ^11^ F. Hoffmann‐La Roche Ltd. Diagnostics Division Basel Switzerland; ^12^ Department of Medical Oncology University of Groningen University Medical Center Groningen Groningen the Netherlands; ^13^ Department of Medical and Surgical Specialties Radiological Sciences and Public Health Institute of Cardiology University of Brescia Brescia Italy; ^14^ Cardiovascular Disease Unit IRCCS Ospedale Policlinico San Martino Genova Italy; ^15^ IRCCS Italian Cardiovascular Network Department of Internal Medicine University of Genova Genova Italy

**Keywords:** heart failure, neoplasms, biomarkers, tumour, natriuretic peptides

## Abstract

**Background:**

There is increasing recognition that heart failure (HF) and cancer are conditions with a number of shared characteristics.

**Objectives:**

To explore the association between tumour biomarkers and HF outcomes.

**Methods:**

In 2,079 patients of BIOSTAT‐CHF cohort, we measured six established tumour biomarkers: CA125, CA15‐3, CA19‐9, CEA, CYFRA 21‐1 and AFP.

**Results:**

During a median follow‐up of 21 months, 555 (27%) patients reached the primary end‐point of all‐cause mortality. CA125, CYFRA 21‐1, CEA and CA19‐9 levels were positively correlated with NT‐proBNP quartiles (all *P* < 0.001, *P* for trend < 0.001) and were, respectively, associated with a hazard ratio of 1.17 (95% CI 1.12–1.23; *P* < 0.0001), 1.45 (95% CI 1.30–1.61; *P* < 0.0001), 1.19 (95% CI 1.09–1.30; *P* = 0.006) and 1.10 (95% CI 1.05–1.16; *P* < 0.001) for all‐cause mortality after correction for BIOSTAT risk model (age, BUN, NT‐proBNP, haemoglobin and beta blocker). All tumour biomarkers (except AFP) had significant associations with secondary end‐points (composite of all‐cause mortality and HF hospitalization, HF hospitalization, cardiovascular (CV) mortality and non‐CV mortality). ROC curves showed the AUC of CYFRA 21‐1 (0.64) had a noninferior AUC compared with NT‐proBNP (0.68) for all‐cause mortality (*P* = 0.08). A combination of CYFRA 21‐1 and NT‐proBNP (AUC = 0.71) improved the predictive value of the model for all‐cause mortality (*P* = 0.0002 compared with NT‐proBNP).

**Conclusions:**

Several established tumour biomarkers showed independent associations with indices of severity of HF and independent prognostic value for HF outcomes. This demonstrates that pathophysiological pathways sensed by these tumour biomarkers are also dysregulated in HF.

## Graphical abstract

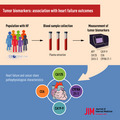

## Introduction

Heart failure (HF) is a devastating medical condition, with increasing prevalence, and despite extensive treatment modalities, mortality remains very high. It has been appreciated that noncardiovascular (CV) mortality in HF is substantial, and over the last decade, a shift has been observed in mode of death, with non‐CV death nowadays being more common than 20 years ago. In particular, cancer in HF is a frequent co‐morbidity, and it is estimated that 5‐25% of all deaths can be attributed to cancer 1, 2, 3. Although at first sight the two diseases may appear in two separate entities, there has been increasing awareness that cancer and HF are conditions with a number of shared characteristics. For instance, classical CV risk factors in fact also predict new‐onset cancer. Furthermore, genetic factors, inflammation and several circulating factors are important for both HF and cancer development 4, 5.

In the prediction of incident cancer or the monitoring of prevalent (or treated) cancers, there is a prominent role for tumour biomarkers 6. For instance, surveillance programmes of colorectal cancer often make use of the tumour biomarker CEA, and for monitoring breast cancer, the tumour biomarker CA15‐3 is in use. Strikingly, several presumed tumour biomarkers, such as CA125 and human epididymis protein 4 (HE4), have been shown to strongly predict outcome in HF as well 7, 8, 9, 10. Given the emerging appreciation that HF and cancer may be two diseases within one spectrum, these observations may actually fit this concept, and tumour biomarkers may broadly signify progression of pathways that classically were linked to certain cancers, but may also be of importance for HF progression.

We hypothesized that tumour biomarkers at large would be correlated with markers of HF severity and would be independently associated with outcomes in HF. We therefore measured six biomarkers that are in use for various cancers, including ovarian, breast, lung, pancreatic, colorectal and germ cell cancer, in 2079 patients of the ‘Systems Biology Study to Tailored Treatment in Chronic Heart Failure’ (BIOSTAT‐CHF) study 11, 12, 13.

## Materials and methods

### Study population

For the present study, HF patients were included from BIOSTAT‐CHF index cohort, which has been described before in detail 11, 12. In summary, The BIOSTAT‐CHF study is a multicenter, observational clinical study performed in 11 European countries. The patients aged ≥ 18 years, with either new‐onset or worsening HF, as defined as left ventricular ejection fraction (LVEF) of ≤ 40% or plasma concentration of brain natriuretic peptide (BNP) >400 ng/L and/or N‐terminal pro‐B‐type natriuretic peptide (NT‐proBNP) >2000 ng/L, were subjected to treatment with furosemide ≥ 40 mg/day or equivalent at the time of inclusion. Meanwhile, patients were not previously treated with angiotensin‐converting enzyme (ACE) inhibitors/angiotensin receptor antagonists (ARBs) and beta blockers, or received ≤ 50% of target doses of these drugs, and were initiated or up‐titrated with these drugs by the treating physician. The BIOSTAT‐CHF complied with the Declaration of Helsinki and was approved by national and local ethics committees (EudraCT 2010‐020808‐29; R&D Ref Number 2008‐CA03; MREC Number 10/S1402/39) 11. Written informed consent was provided by the patients enrolled in the study. In total, 2516 patients were included in the BIOSTAT‐CHF index cohort and plasma samples of 2079 patients were available for tumour biomarker analysis.

### Tumour biomarker measurement

The tumour biomarkers CA125, CA15‐3, CA19‐9, CEA, CYFRA 21‐1, AFP, alongside with the HF marker (NT‐proBNP) were assessed in venous blood samples. Blood samples were centrifuged for 15 min at 2500 g at 4°C, and afterwards, plasma was collected and stored at −80°C until further analysis. All six tumour biomarkers were measured by the Roche Elecsys^®^ assay on a cobas e 411 analyzer using standard methods (Roche Diagnostics GmbH, Mannheim, Germany). This platform allows to quantitatively measure human CA125, CA15‐3, CA19‐9, CEA, CYFRA 21‐1, and AFP levels in plasma with high sensitivity.

### Study end‐points

The associations between tumour biomarkers and clinical outcomes were evaluated. We considered all‐cause mortality as the primary end‐point. Secondary end‐points included composite of all‐cause mortality and HF hospitalization, HF hospitalization, CV mortality, and non‐CV mortality.

### Statistical analyses

Data are presented as means ± standard deviation (SD) when data were normally distributed, or as medians with interquartile ranges (IQR), when data were non‐normally distributed. Continuous normally distributed variables were compared using Student’s independent t‐test or ANOVA, whereas skewed variables were compared using Mann–Whitney U‐test or Kruskal–Wallis H‐test. The distribution of tumour biomarker levels was observed according to NT‐proBNP levels (ng/L) quintiles. Trends of the tumour biomarkers over NT‐proBNP quintiles were statistically tested with an extension of the Wilcoxon rank‐sum test. The assumption of normal distribution was checked before linear regression analysis. If necessary, skewed variables were log‐transformed (using natural logarithm). Univariable and multivariable regression analyses were conducted to analyse the association between tumour biomarkers and variables, in which all variables associated with tumour biomarkers with *P* < 0.10 in univariable analysis were included in multivariable regression models and were subjected to the backward elimination method.

Cox proportional hazard regression analysis was performed for all end‐points to evaluate the independent prognostic value of each tumour biomarker and NT‐proBNP. All‐cause mortality, HF hospitalization and the composite end‐point of both were corrected for their respective BIOSTAT risk model (including age, blood urea nitrogen [BUN], NT‐proBNP, haemoglobin and beta blocker use at baseline), as previously published 12, 14. The models for CV mortality were corrected for age, BUN, NT‐proBNP, troponin T and sodium, while non‐CV mortality models included age, haemoglobin, C‐reactive protein and history of malignancy. These correction factors were selected using a regression model, including all factors that were univariably significantly associated with CV and non‐CV mortality, respectively. Receiver operating characteristic (ROC) curve analysis was used to determine the predictive performance of each tumour biomarker. The area under the curve (AUC) was calculated as the diagnostic measure of the test. A 2‐tailed *P*‐value < 0.05 was considered to denote statistically significant differences, while for interaction testing a *P*‐value < 0.1 was used. For our primary and prespecified secondary analyses, we present Bonferroni‐corrected *P*‐values. The non‐prespecified, exploratory secondary analyses in the supplement were not corrected, and these data should be considered hypothesis‐generating but not definitive. Data analysis was performed with R (version 3.5.1, R Foundation for Statistical Computing, Vienna, Austria) and Stata15.1 (StataCorp, 2017, College Station, TX, USA: StataCorp LLC).

## Results

### Baseline characteristics of patients

The study included 2079 of 2516 (83%) patients enrolled in BIOSTAT‐CHF because of plasma sample availability and the limit of detection of the assay used. The baseline characteristics of these patients are presented in Table 1, which is comparable with the whole BIOSTAT‐CHF index cohort as shown in Table S1. The mean age (± SD) of this study population was 69 ± 12 years and 26.3% were female. Median LVEF (+ IQR) was 30% (25–36), recorded mostly by echocardiography. Median NT‐proBNP value (+ IQR) was 2696 (1204–5671).

**Table 1:** Baseline characteristics of study population

Factor	Value (*N* = 2079)
Clinical characteristics	
Age, years	69 ± 12
Sex, Females, *n* (%)	547 (26.3)
BMI, kg/m^2^	27.9 ± 5.5
Ischaemic aetiology, *n* (%)	920 (44.3)
NYHA functional class III/IV, *n* (%)	1237 (59.5)
LVEF, %	30 (25–36)
HFrEF, *n* (%)	1502 (72.2)
HFmrEF, *n* (%)	232 (11.2)
HFpEF, *n* (%)	125 (6.0)
Oedema, *n* (%)	1017 (48.9)
Systolic blood pressure, mmHg	124 ± 22
Heart rate, b.p.m	80 ± 20
Hospitalization, type of visit, *n* (%)	
Scheduled outpatient clinic	507 (24.4)
Unscheduled outpatient clinic	95 (4.6)
Inpatient hospitalisation	1477 (71.0)
Previous HF hospitalization in last year, *n* (%)	646 (31.1)
Laboratory	
NT‐proBNP, ng/L	2696 (1204–5671)
Troponin T, µg/L	31.5 (19.2–53.5)
Haemoglobin, g/dL	13.2 ± 1.9
Sodium, mmol/L	140 (137–142)
Potassium, mmol/L	4.2 (3.9–4.6)
eGFR, mL/min/1.73 m^2^	60 (44–77)
Albumin, g/L	32 ± 9
BUN, mmol/L	11.1 (7.4–17.9)
CRP, mg/L	13 (6–27)
HDL, mmol/L	1.0 (0.8–1.3)
IL‐6, pg/mL	5.2 (2.8–10.2)
Leucocytes, 10^9^/L	7.8 (6.4–9.6)
ASAT, U/L	25 (19–35)
ALAT, U/L	25 (16–37)
γ‐GT, U/L	54 (28–106)
Alkaline phosphatase, µg/L	84 (65–117)
Medical history	
Atrial fibrillation, *n* (%)	949 (45.6)
Diabetes mellitus, *n* (%)	660 (31.7)
COPD, *n* (%)	353 (17.0)
Renal disease, *n* (%)	592 (28.5)
Malignancy, *n* (%)	81 (3.9)
Smoking, *n* (%)	
Never	755 (36.3)
Past	1015 (48.8)
Current	306 (14.7)
Device therapy, *n* (%)	498 (24.0)
Medication, *n *(%)	
Loop diuretics	2070 (99.6)
Beta blockers	1731 (83.3)
ACE inhibitors/ARB	1490 (71.7)
MRA	1097 (52.8)
Oral anticoagulants	803 (38.6)

ACE, angiotensin‐converting enzyme; ALAT, alanine transaminase; ARB, angiotensin receptor blocker; ASAT, aspartate transaminase; BMI, body mass index; BUN, blood urea nitrogen; COPD, chronic obstructive pulmonary disease; CRP, C‐reactive protein; eGFR, estimated glomerular filtration rate; γ‐GT, γ‐glutamyl transpeptidase; HDL, high‐density lipoprotein; HFmrEF, heart failure with mid‐range ejection fraction; HFpEF, heart failure with preserved ejection fraction; HFrEF, heart failure with reduced ejection fraction; IL‐6, interleukin 6; LVEF, left ventricular ejection fraction; NT‐proBNP, N‐terminal pro‐B‐type natriuretic peptide; NYHA, New York Heart Association; MRA, mineralocorticoid receptor antagonist.

### Levels of tumour biomarkers: reference values, values in BIOSTAT‐CHF

CA125, CA15‐3, CA19‐9, CEA, CYFRA 21‐1 and AFP are generally regarded as biomarkers for ovarian, breast, pancreatic, colon, lung and germ cell cancer, respectively 6. As Table S2 signified, the measurements of these six tumour biomarkers in BIOSTAT cohort were obtained. The normal reference values were based on literature published before 15, 16, 17, 18, 19. The exact number of assessments for each tumour biomarker was as follows: CA125 (*N* = 2069), CA15‐3 (*N* = 2073), CA19‐9 (*N* = 2066), CEA (*N* = 2079), CYFRA 21‐1 (*N* = 2054) and AFP (*N* = 2078).

There were 81 patients with a history of cancer, and tumour biomarker levels were comparable between these patients and the patients without malignancy, although there were suggestive differences in CA125 and CYFRA 21‐1, as shown in Table S3. Given that there were 46% patients with atrial fibrillation (AF), which is known to increase cardiac biomarkers including cardiac troponin and NT‐proBNP 20, 21, we additionally studied the tumour biomarker levels, stratified by the absence of AF. As presented in Table S4, levels of CA125 and CA19‐9 were significantly higher in the patients with a history of AF.

### Distribution of tumour biomarker levels across NT‐proBNP quintiles

Patients were categorized according to NT‐proBNP quintiles as depicted in Table 2. With increasing NT‐proBNP levels, the plasma concentrations of CA125, CA19‐9, CEA and CYFRA 21‐1 significantly increased (all *P* < 0.001; *P* for trend < 0.001). However, there were no clear associations between increasing NT‐proBNP and CA15‐3 or AFP.

**Table 2:** Interaction between tumour markers and NT‐proBNP in HF

Factor	Quintiles of NT‐proBNP					*P*‐value^a^	*P‐*value for trend^a^
	Quintile 1 ≤ 984 ng/L	Quintile 2 985‐2010 ng/L	Quintile 3 2011‐3617 ng/L	Quintile 43618‐6905 ng/L	Quintile 5 ≥ 6906 ng/L		
CA125 (U/mL), median (IQR)	14.0 (9.1–24.5)	27.4 (13.4–71.2)	43.4 (16.5–107.2)	57.4 (21.6–123.8)	70.0 (32.4–168.5)	<0.001	<0.001
CA15‐3 (U/mL), median (IQR)	19.7 (14.4–25.5)	20.4 (14.7–26.4)	20.0 (13.9–26.7)	18.4 (13.2–24.9)	20.0 (14.9–28.8)	0.23	1.0
CA19‐9 (U/mL), median (IQR)	8.1 (4.5–14.5)	9.6 (5.2–17.7)	10.8 (6.7–19.7)	10.4 (5.8–19.7)	13.6 (6.9–23.9)	<0.001	<0.001
CEA (ng/mL), median (IQR)	2.2 (1.5–3.2)	2.4 (1.5–3.3)	2.3 (1.6–3.5)	2.7 (1.8–4.1)	2.8 (1.8–4.3)	<0.001	<0.001
CYFRA 21‐1 (ng/mL), median (IQR)	1.9 (1.4–2.7)	2.0 (1.4–2.7)	1.9 (1.4–2.8)	2.1 (1.5–2.8)	2.5 (1.7–3.5)	<0.001	<0.001
AFP (IU/mL), median (IQR)	1.9 (1.2–2.9)	1.8 (1.1–3.1)	1.9 (1.1–2.9)	1.8 (1.2–2.9)	1.7 (1.0–2.7)	1.0	0.26

^a^ Bonferroni‐adjusted *P*‐values.

### Tumour biomarkers and outcomes in HF

#### Primary outcome

During a median follow‐up of 21 months, 555 (27%) patients reached the primary outcome of all‐cause mortality, as shown in Table 3. Compared with the patients alive, those who died had significantly higher levels of CA125, CA15‐3, CA19‐9, CEA and CYFRA 21‐1 (all *P* < 0.001). Levels of AFP were comparable between alive and deceased patients. There were significant associations between NT‐proBNP and CA125, NYHA class III/IV and CA19‐9 as displayed in Table S5, also after correction with the multivariable models (both *P* < 0.05).

**Table 3:** Correlation between tumour marker levels and mortality

Factor	Patients who survived	Patients who died	*P‐*value^a^
N	1524	555	<0.001
CA125 (U/mL)	27.1 (13.3–83.8)	55.5 (21.1–137.5)	<0.001
CA15‐3 (U/mL)	19.4 (13.7–25.7)	20.7 (15.0–29.1)	<0.001
CA19‐9 (U/mL)	9.2 (5.4–17.1)	13.1 (7.4–24.6)	<0.001
CEA (ng/mL)	2.3 (1.5–3.5)	2.8 (1.9–4.1)	<0.001
CYFRA 21‐1 (ng/mL)	1.9 (1.4–2.7)	2.5 (1.8–3.7)	<0.001
AFP (IU/mL)	1.8 (1.2–2.9)	1.7 (1.0–2.7)	0.11

^a^ Bonferroni‐adjusted *P*‐values.

**Fig. 1:**
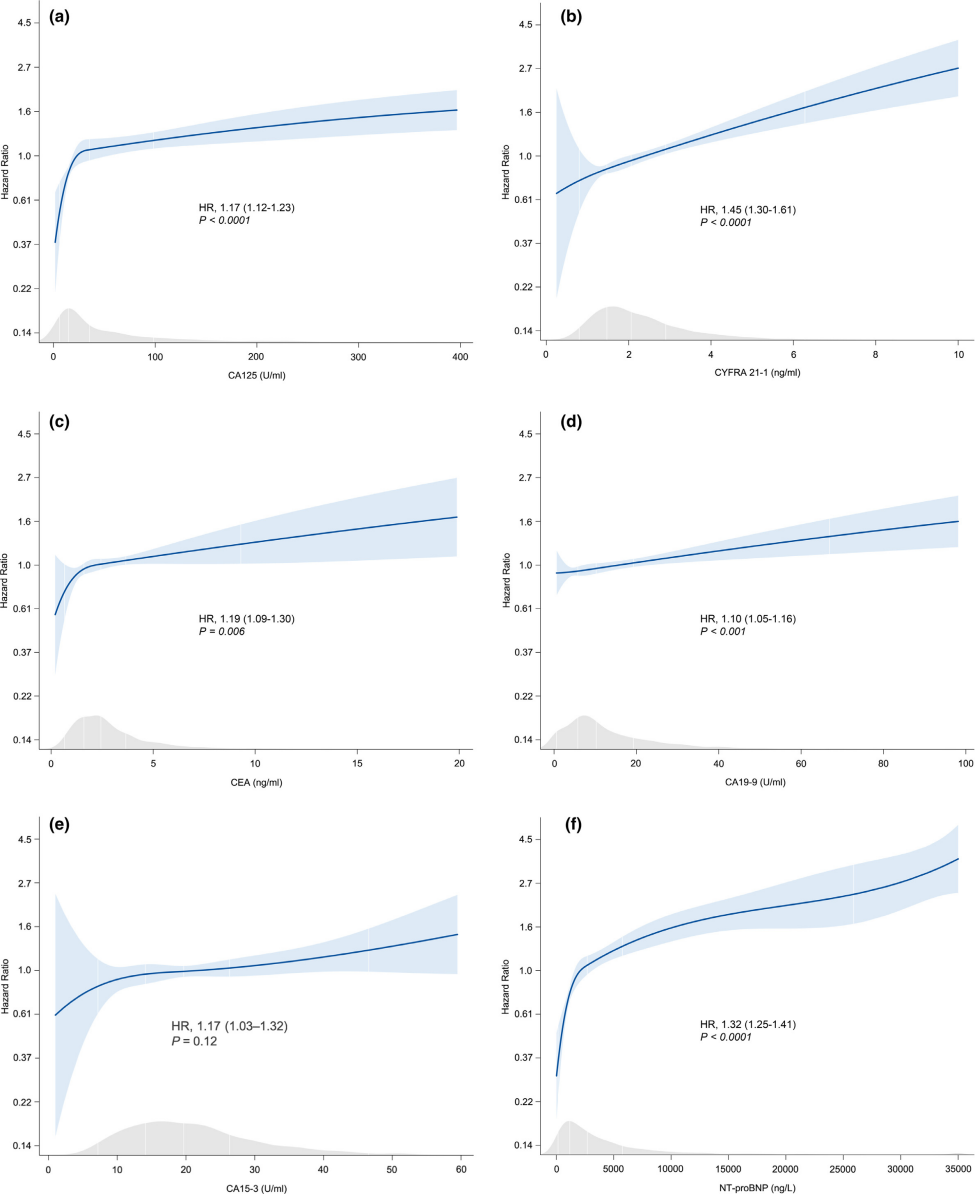
Cox regression models, estimating the predictive value of tumour markers and NT‐proBNP for all‐cause mortality. (a) CA125. (b) CYFRA 21‐1. (c) CEA. (d) CA19‐9. (e) CA15‐3. (f) NT‐proBNP. The *P*‐values presented are Bonferroni‐adjusted. HR: hazard ratio. Numbers between brackets represent the 95% CI. All models are corrected for the BIOSTAT risk model (including age, BUN, NT‐proBNP (except for NT‐proBNP), haemoglobin and beta blocker use at baseline).

Adjusted Cox regression splines for all‐cause mortality demonstrated the associations between tumour biomarkers and mortality (displayed in Fig. 1). Circulating levels of CA125, CYFRA 21‐1, CEA and CA19‐9 correlated with respective hazard ratios (HR) of 1.17 (95% CI 1.12–1.23; *P* < 0.0001), 1.45 (95% CI 1.30–1.61; *P* < 0.0001), 1.19 (95% CI 1.09–1.30; *P* = 0.006) and 1.10 (95% CI 1.05–1.16; *P* < 0.001) for all‐cause mortality, after correction for age, BUN, NT‐proBNP, haemoglobin and beta blocker use. In comparison, the HR of NT‐proBNP for all‐cause mortality was 1.32 (95% CI, 1.25–1.41; *P* < 0.0001). There were no significant relations between mortality and CA15‐3 or AFP.

#### Secondary outcomes

We used multivariable‐adjusted Cox regression models to separately analyse predictive value of each tumour biomarker for the secondary end‐points: the composite of all‐cause mortality and HF hospitalization (*N* = 870), HF hospitalization (*N* = 525), CV mortality (*N* = 368) and non‐CV mortality (*N* = 110). All tumour biomarkers except AFP had significant associations with one or more secondary end‐points after correction for respective risk factors as shown in Table S6. Only CA125 was strongly associated with HF hospitalization risk (HR, 1.10; 95% CI 1.05–1.15; *P* < 0.001). CA125, CYFRA 21‐1 and CA19‐9 were significantly associated with both CV and non‐CV mortality. There was a remarkable association between CEA and CV mortality (HR, 1.18; 95% CI, 1.06–1.32; *P* = 0.003).

### Comparison of tumour biomarkers with NT‐proBNP to predict mortality in HF

**Fig. 2:**
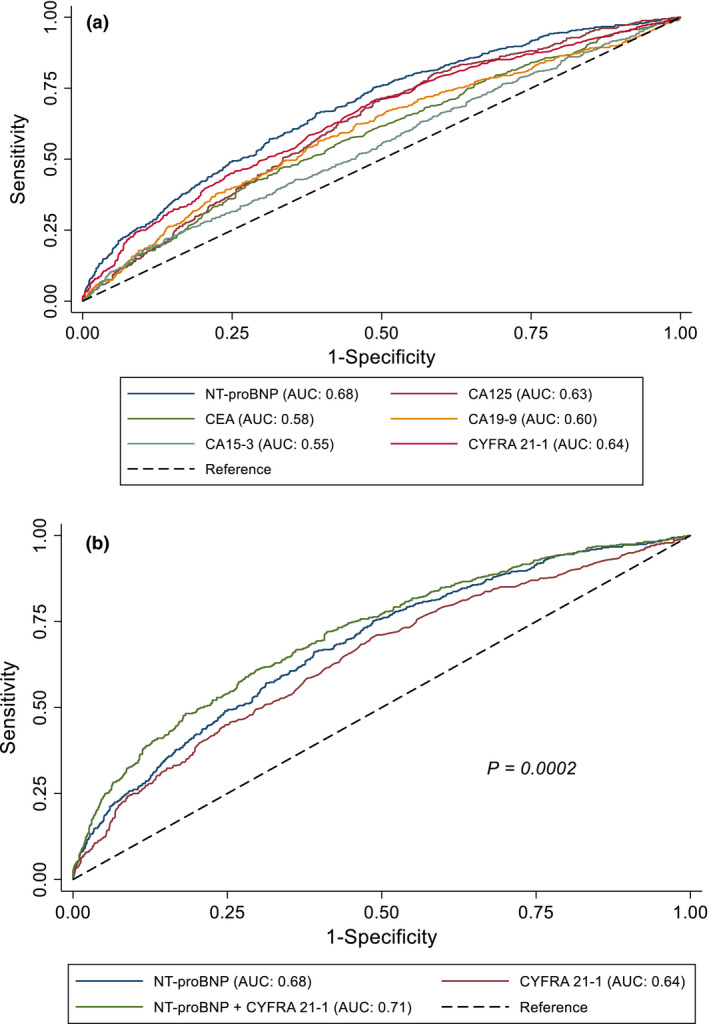
(a) ROC curves for CA125, CYFRA 21‐1, CEA, CA19‐9, CA15‐3 and NT‐proBNP for all‐cause mortality. (b) ROC curve for NT‐proBNP, CYFRA 21‐1 and both biomarkers combined for all‐cause mortality. *P*‐value (Bonferroni‐corrected) refers to the comparison between NT‐proBNP and NT‐proBNP + CYFRA 21‐1.

To provide better insight into the predictive value of the tumour biomarkers for all‐cause mortality, we benchmarked them against the established HF biomarker NT‐proBNP. ROC curves were designed to further compare the value of different tumour biomarkers to NT‐proBNP in predicting the primary outcome as displayed in Fig. 2a. The AUCs for CA125, CYFRA 21‐1, CEA, CA15‐3 and CA19‐9 were 0.63 (95% CI 0.59–0.64), 0.64 (95% CI 0.62–0.67), 0.58 (95% CI 0.55–0.61), 0.55 (95% CI 0.53–0.58) and 0.60 (95% CI 0.57–0.63), respectively, while the AUC of NT‐proBNP was 0.68 (95% 0.65–0.71).

Only CYFRA 21‐1 had a comparable value of AUC with NT‐proBNP (0.64 versus 0.68; Bonferroni *P* = 0.08). The AUC of combined NT‐proBNP and CYFRA 21‐1 was 0.71 (95% CI, 0.69–0.74), significantly higher than the models with either marker alone (*P* = 0.0002 compared with NT‐proBNP) as shown in Fig. 2b. The other tumour biomarkers did not show additional predictive value on top of NT‐proBNP in the ROC analysis.

## Discussion

Recent studies have suggested that cancer and HF share risk factors, including age, sex, smoking, genetics, obesity, diabetes mellitus and hypertension, and pathophysiological pathways such as inflammation and oxidative stress to a large extent, and a provocative view is that these two diseases are two manifestations of the same disease spectrum 1, 4, 5, 22, 23, 24, 25. In an unorthodox fashion, we therefore in this study aimed to provide evidence for this hypothesis, by evaluating the prognostic value and clinical correlates of six commonly used tumour biomarkers in a large, well‐defined cohort of HF patients. First, we demonstrate that four out of six tumour biomarkers have independent prognostic value and predict all‐cause mortality. We further show that several tumour biomarkers are strongly related to markers of HF severity, including NT‐proBNP and NYHA class. Finally, we show that CYFRA 21‐1 had equivalent predictive utility for all‐cause mortality compared with NT‐proBNP. We conclude that the very pathways and pathological signals that are ‘sensed’ by tumour biomarkers are present in HF and have relationships with HF severity and outcomes.

### Tumour biomarkers

We measured biomarkers that are referred to as tumour biomarkers, and as such may be used by clinicians. In reality, the sensitivity and specificity of the markers have been debated 26, 27. Further, it is well recognized that none of these markers are specific for any particular tumour, and several confounding factors have been identified. For instance, CYFRA 21‐1 levels are increased in smokers 28. Although our models were adjusted, we acknowledge there will be residual confounding that we cannot account for. However, zooming in on the described biology of the biomarkers, it is intriguing that none of the markers have appreciable cardiac production. Indeed, the relations between the tumour biomarkers must be indicative of systemic manifestations of the HF syndrome.

Several tumour biomarkers have been previously studied in HF before, but our study is more comprehensive and adequately powered. CA125, which is recognized as a tumour biomarker of ovarian cancer, has been studied in HF before. CA125 is produced by serous epithelial cells and mesothelial cells as a response to congestion and inflammatory stimuli, triggered by increasing tumour necrosis factor‐alpha (TNF‐α) and IL‐4 29, 30. Recent studies have shown the prognostic role of CA125 in different cardiac diseases 29, 31, 32. Increased levels of CA125 have been reported in patients with atrial fibrillation, HF and acute myocardial infarction, which might be induced by mechanical stress and inflammation 31, 33, 34, 35. CA125 levels have been associated with the risk of adverse outcomes of HF and have been put forward as a promising biomarker for guiding HF therapy 8, 36. Interestingly, another biomarker advocated for epithelial ovarian cancer (EOC) is HE4, which is currently used to monitor recurrence and plays a role in molecular pathways related to tumour proliferation and metastasis 37, 38. Likewise, recent clinical data indicated that HE4 levels are strongly associated with HF and chronic kidney disease (CKD) severity, and independently predict HF outcomes 9, 10, 39. Further, plasma levels of CA19‐9, a marker for pancreatic cancer and secreted by tumour tissues and normal biliary epithelium, were shown to be higher in HF patients compared to healthy controls 40, 41, 42.

In addition, other common tumour biomarkers in clinical use were also measured in our study. CYFRA 21‐1, a cytokeratin, is a sensitive marker for non‐small‐cell lung cancer (NSCLC, 85% of lung cancer), expressed in simple epithelium, including bronchial epithelium, and in cancers derived from those cells 43, 44. CA15‐3, a member of MUC1 family, is produced by normal simple epithelial cells lymphocytes, dendritic cells and a variety of carcinomas and mainly used as a marker for breast cancer, which participates in cell repair and survival 45. Additionally, CEA is regarded as a biomarker of colon cancer and involved in cancer invasion and metastasis, mostly expressed in cancer cells and normally produced in gastrointestinal tissue during foetal development 46. Finally, AFP mainly synthesized by the foetal yolk sac and liver during embryonic development is a marker for germ cell tumours 47. This study is the first to systematically analyse the levels of all these markers with regard to clinical correlates, severity and outcomes of HF in a large well‐defined cohort, despite some researches carried out in small numbers (35‐191) of patients 40, 48.

### Tumour biomarkers are associated with indices of HF severity

We demonstrated strong relationships with established indices of HF severity for four out of six tumour biomarkers. All biomarkers (except AFP) had linear relations with each increase in NT‐proBNP quintile. A significant correlation was found between NT‐proBNP and CA125, also after correction with the multivariable models, in line with previous reports that CA125 relates to congestion, and congestion is strongly linked to pulmonary and peripheral oedema, which in turn is the strongest determinant of worse functional class in HF 8. Strikingly, CA15‐3 did not increase with NT‐proBNP, and its levels appeared to independently from behave NT‐proBNP levels. AFP did not substantially change with any of the HF clinical characteristics. AFP is different from the other markers, and its production is mediated by germ cells, and not surprisingly, levels were much higher in younger patients. Our data do not show any relation between AFP and HF.

### Tumour biomarkers are prognostic factors in HF

Cox regression and ROC analysis were conducted as observed, and four out of six tumour biomarkers, CA15‐3 and AFP being the exception, were independent predictors of all‐cause mortality. We used a previously published multivariable model, comprising of age, BUN, NT‐proBNP, haemoglobin and beta blocker, with a Harrell’s C‐statistic of 0.69 for all‐cause mortality 12. On top of this model, CA125, CYFRA 21‐1, CEA and CA19‐9 independently predicted all‐cause mortality. Additionally, ROC analysis showed that only CYFRA 21‐1 had an AUC comparable to NT‐proBNP for all‐cause mortality. A model consisting of NT‐proBNP and CYFRA 21‐1 had the best AUC (0.71), which was significantly higher than univariable models comprising NT‐proBNP or CYFRA 21‐1. Strikingly, the prognostic metrics were numerically comparable for all‐cause mortality, CV mortality and non‐CV mortality. The number of non‐CV deaths was small (*N* = 110), and cancer‐related deaths were not stringently adjudicated, but likely, a large proportion of the non‐CV death was due to cancer. From these exploratory analyses, we conclude that elevated levels of tumour biomarkers are as strongly associated with CV mortality as they are with non‐CV mortality, again hinting towards the interaction between HF and cancer.

### Scientific and clinical impact

Importantly, based on our results, we – by no means – advocate the use of tumour biomarkers for HF risk prediction, rather, we defer from it further proof that the biological pathways that are sensed by these tumour biomarkers are perturbed in HF. Our data thus in our opinion provide further ground for the growing notion that there is an interplay between cancer and HF 1, 2, 4. Triggers and pathways that are operative in cancer, and that control the production or release of tumour biomarkers, apparently are also present in HF, with increases of the same markers as a consequence. Whether or not these triggers are indicative of a precancerous state is not proven. We and other researchers have recently observed that the presence of HF is associated with an increased incidence of cancer 49, 50. As a result, a disproportionate percentage of HF patients die of cancer 1, 2. We of course cannot exclude that a proportion of HF patients with elevations in tumour markers in fact had (undiagnosed) cancer, in other words, that the tumour marker levels indeed were reflective of underlying cancer. Based on the fact that a diagnosis of cancer is relatively uncommon in patients with prevalent heart failure and that a limited number of clinical decisions are based on biomarker results 22, future studies should address the specific value of tumour biomarker elevations in patients with HF with regard to cancer screening, diagnosis and treatment. Collectively, we believe that our study provides compelling data that underscore the intimate and reciprocal relation between cancer and HF.

### Strengths and limitations

The novelty of our study is the measurement of six tumour biomarkers in a well‐defined sizeable cohort (BIOSTAT‐CHF), in which CA125, CYFRA 21‐1, CEA and CA19‐9 provided equivalent predictive value with NT‐proBNP for clinical HF outcomes. For the first time, we were able to demonstrate that a combination of traditional HF marker (NT‐proBNP) with established marker (CYFRA 21‐1) for oncological disease provided a stronger predictive value on clinical outcome than the individual markers were capable of. Moreover, we confirmed that in patients with HF, outcomes can also be predicted when based on tumour biomarkers, strengthening the hypothesis that HF and cancer may share a substantial communality in underlying disease‐modifying mechanisms. Our results are based on a multicenter, multinational cohort with large clinical and biochemical parameters and can be a good reflection of contemporary European HF patients. Meanwhile, we constructed different analytical methods to correct for indication bias, which strengthened the stability and reliability of the results.

There are limitations to the current study. First of all, the presence of cancer at baseline was not consistently  studied (e.g. by imaging studies), and follow‐up was mainly aimed to describe CV events and mortality, while cancer events and mortality were not stringently adjudicated. Further, BIOSTAT‐CHF is mainly an European Caucasian cohort, which makes it difficult to extend the results to other ethnicities and populations beyond Europe. The majority of patients were males, and less statistical power and independent determinants were available to make statements about females. Similarly, our findings are mainly based on patients with decompensated HF with reduced ejection fraction (72%) and may thus not apply to other types of HF. In addition, only six common tumour biomarkers were measured due to the limit of Elecsys^®^ assay, and the dynamic changes over time of the tumour biomarkers cannot be detected due to such a single‐time study. Moreover, there were 437 samples missing, due to low sample volume or because of limit of detection, so that not all tumour markers for each sample could be measured. Further, pathophysiological and biological mechanisms can clearly not be studied directly given the observational character of the BIOSTAT study. Finally, although results were adjusted by a published set of variables, residual bias and confounding cannot be excluded, despite multiple statistical techniques aimed to provide proper correction.

## Conclusion

Our results fuel the notion that cancer and HF are two manifestations of the same disease continuum. Several commonly used tumour biomarkers are related to HF severity and have independent predictive values for HF outcomes, which further supports there is an interplay between HF and cancer. Elevated levels of tumour biomarkers can indicate worse outcomes, in the context of HF presence or absence.

## Conflict of interest

The UMCG, which employs several of the authors, has received research grants and/or fees from AstraZeneca, Abbott, Bristol‐Myers Squibb, Novartis, Novo Nordisk and Roche. RAdB received personal fees from Abbott, AstraZeneca, Novartis, and Roche. SDA reports personal fees from Vifor Int, Bayer, Boehringer Ingelheim, Novartis, Servier, Respicardia and Impulse Dynamics outside the submitted work; and grants from Abbott Vascular and Vifor Int outside the submitted work. JGC reports personal fees from Amgen, AstraZeneca, Bayer, Bristol‐Myers Squibb, GlaxoSmithKline, Medtronic, MyoKardia, Novartis International AG, Philips, Sanofi, Servier, Stealth Biopharmaceuticals, Torrent Pharmaceuticals, Vifor and Abbott; grants from Amgen, Bayer, Bristol‐Myers Squibb, Medtronic, Novartis, Pharmacosmos, Pharma Nord, Stealth Biopharmaceuticals, Torrent Pharmaceuticals and Vifor; and nonfinancial support from Pharmacosmos, Pharma Nord, Vifor, Novartis International AG and Medtronic. CCL reports grants from AstraZeneca, Amgen, Merck & Co, Novartis and Servier, during the conduct of the study. PLvH is an employee of Hoffmann‐La Roche Ltd and holds stock in this company. MM reports grants from European Community during the conduct of the study and personal fees from Bayer, Novartis and Servier outside the submitted work. PA reports personal fees from Servier, Novartis, Boehringer Ingelheim, Daiichi Sankyo, Bayer, Pfizer, Menarini, Sanofi, MSD and GSK as well as an investigator‐initiated grant from Boehringer Ingelheim. AAV reports grants from European Commission during the conduct of the study and Roche Diagnostics outside the submitted work; personal fees from Amgen, Boehringer Ingelheim, AstraZeneca, Bayer, Cytokinetics, GlaxoSmithKline, MyoKardia, Roche Diagnostics, Novartis and Servier, outside the submitted work. All other authors declare no competing interests.

## Supporting information


**Table S1.** Baseline characteristics of study population and patients in BIOSTAT‐CHF index cohort.
**Table S2.** Levels of tumor biomarkers: reference values, values in malignancies and values in BIOSTAT‐CHF.
**Table S3.** Differences in median tumor biomarker levels based on history of malignancy.
**Table S4.** Differences in median tumor biomarker levels based on history of atrial fibrillation.
**Table S5.** Linear regression models for tumor biomarkers.
**Table S6.** Cox regression analysis for secondary outcomes.
