# Supplementary material for: Tumour biomarkers: association with heart failure outcomes
Source: J Intern Med. 2020 May 5;288(2):207–18. doi: 10.1111/joim.13053 (PMC7496322; doi:10.1111/joim.13053)
Supplement: Supplementary file 1 — Table S1. Baseline characteristics of study population and patients in BIOSTAT‐CHF index cohort. Table S2. Levels of tumor biomarkers: reference values, values in malignancies and values in BIOSTAT‐CHF. Table S3. Differences in median tumor biomarker levels based on history of malignancy. Table S4. Differences in median tumor biomarker levels based on history of atrial fibrillation. Table S5. Linear regression models for tumor biomarkers. Table S6. Cox regression analysis for secondary outcomes. [file JOIM-288-207-s001.docx]

**Table S1** Baseline characteristics of study population and patients in BIOSTAT-CHF index cohort

| Factor | Study population (N=2079) | Patients in BIOSTAT-CHF index cohort (N=2516) |
| --- | --- | --- |
| Clinical characteristics |  |  |
| Age, years | 69 ± 12 | 69 ± 12 |
| Sex, Females, n (%) | 547 (26.3%) | 670 (26.6%) |
| BMI, kg/m^2^ | 27.9 ± 5.5 | 27.9 ± 5.5 |
| NYHA functional class III/IV, n (%) | 1237 (59.5%) | 1522 (60.5%) |
| LVEF, % | 30 (25–36) | 30 (25–36) |
| Systolic blood pressure, mmHg | 124 ± 22 | 125 ± 22 |
| Heart rate, b.p.m | 80 ± 20 | 80 ± 20 |
| Hospitalization, type of visit, n (%) |  |  |
| - Outpatient clinic | 602 (29.0%) | 822 (32.7%) |
| - Inpatient hospitalisation | 1477 (71.0%) | 1694 (67.3%) |
| Previous HF hospitalization in last year, n (%) | 646 (31.1%) | 794 (31.6%) |
| Laboratory |  |  |
| NT-proBNP, ng/L | 2696 (1204–5671) | 2698 (1178–5755) |
| Troponin T, µg/L | 31.5 (19.2–53.5) | 31.3 (19.0–53.8) |
| Sodium, mmol/L | 140 (137–142) | 140 (137–142) |
| Hemoglobin, g/dL | 13.2 ± 1.9 | 13.2 ± 1.9 |
| Potassium, mmol/L | 4.2 (3.9–4.6) | 4.2 (3.9–4.6) |
| eGFR, mL/min/1.73 m^2^ | 60 (44–77) | 67.1 (50–86) |
| BUN, mmol/L | 11.1 (7.4–17.9) | 11.1 (7.4–17.7) |
| Medical history |  |  |
| Atrial fibrillation, n (%) | 949 (45.6%) | 1143 (45.4%) |
| Diabetes mellitus, n (%) | 660 (31.7%) | 819 (32.6%) |
| COPD, n (%) | 353 (17.0%) | 436 (17.3%) |
| Medication, n (%) |  |  |
| Loop diuretics | 2070 (99.6%) | 2504 (99.5%) |
| Beta-blockers | 1731 (83.3%) | 2093 (83.2%) |
| ACE-inhibitors/ARB | 1490 (71.7%) | 1820 (72.3%) |
| MRA | 1097 (52.8%) | 1339 (53.2%) |

ACE: angiotensin converting enzyme; ARB: angiotensin receptor blocker; BMI: body mass index; BUN: blood urea nitrogen; COPD: chronic obstructive pulmonary disease; eGFR: estimated glomerular filtration rate; LVEF: left ventricular ejection fraction; NT-proBNP: N-terminal pro-B-type natriuretic peptide; NYHA: New York Heart Association; MRA: mineralocorticoid receptor antagonist

**Table S2** Levels of tumor biomarkers: reference values, values in malignancies and values in BIOSTAT-CHF

| Tumor biomarkers | Malignancies | Reference value | Median value in BIOSTAT (+ IQR) | Number of measurements in BIOSTAT |
| --- | --- | --- | --- | --- |
| CA125 (U/mL) | Ovarian cancer | <35 | 33.7 (14.4–97.2) | 2069 |
| CA15-3 (U/mL) | Breast cancer | <30 | 19.7 (14.1–26.4) | 2073 |
| CA19-9 (U/mL) | Pancreatic cancer | <37 | 10.2 (5.8–19.1) | 2066 |
| CEA (ng/mL) | Colon carcinoma | <5 | 2.4 (1.6–3.7) | 2079 |
| CYFRA 21-1 (ng/mL) | Lung cancer | ≤3.3 | 2.1 (1.5–2.9) | 2054 |
| AFP (IU/mL) | Germ cell cancer | <15 or <8.4 ng/ml | 1.8 (1.1–2.9) | 2078 |

**Table S3** Differences in median tumor biomarker levels based on history of malignancy

| History of malignancy | No (N=1998) | Yes (N=81) | *P-*value |
| --- | --- | --- | --- |
| CA125 (U/mL), median (IQR) | 33.0 (14.3–96.2) | 53.2 (19.9–113.3) | 0.031 |
| CA15-3 (U/mL), median (IQR) | 19.7 (14.1–26.4) | 20.3 (15.7–26.1) | 0.35 |
| CA19-9 (U/mL), median (IQR) | 10.2 (5.7–19.0) | 10.8 (7.1–20.6) | 0.27 |
| CEA (ng/mL), median (IQR) | 2.4 (1.6–3.7) | 2.5 (1.6–3.8) | 0.75 |
| CYFRA 21-1 (ng/mL), median (IQR) | 2.0 (1.5–2.9) | 2.3 (1.8–3.3) | 0.017 |
| AFP (IU/mL), median (IQR) | 1.8 (1.1–2.9) | 1.9 (1.1–2.6) | 0.72 |

**Table S4** Differences in median tumor biomarker levels based on history of atrial fibrillation

| History of atrial fibrillation | No (N=1130) | Yes (N=949) | P-value (Bonferroni corrected) |
| --- | --- | --- | --- |
| CA125 (U/mL), median (IQR) | 30.4 (13.2 – 93.9) | 39.9 (16.3 – 98.6) | 0.006 |
| CA15-3 (U/mL), median (IQR) | 19.5 (14.1 – 25.8) | 20.1 (14.1 – 27.2) | 1.00 |
| CYFRA (ng/mL), median (IQR) | 2.0 (1.4 – 2.9) | 2.1 (1.5 – 2.9) | 0.90 |
| CEA (ng/mL), median (IQR) | 2.3 (1.6 – 3.6) | 2.5 (1.7 – 3.8) | 0.12 |
| CA19-9 (U/mL), median (IQR) | 9.2 (5.4 – 17.5) | 11.3 (6.3 – 21.2) | <0.001 |
| AFP (IU/mL), median (IQR) | 1.8 (1.1 – 2.9) | 1.8 (1.1 – 2.8) | 1.00 |

**Table S5** Linear regression models for tumor biomarkers

|  | Univariable | | Multivariable | |
| --- | --- | --- | --- | --- |
|  | Coefficient | *P*-value | Coefficient | *P*-value |
| CA125 |  |  |  |  |
| Age | - 0.000 | 0.845 |  |  |
| Sex | - 0.044 | 0.488 |  |  |
| BMI | - 0.018 | 0.000 |  |  |
| Ischemic etiology | - 0.121 | 0.029 | - 0.297 | 0.017 |
| NYHA class III/IV | +0.552 | 0.000 |  |  |
| LVEF | - 0.009 | 0.001 |  |  |
| Oedema | +0.712 | 0.000 | +0.309 | 0.017 |
| Systolic blood pressure | - 0.006 | 0.000 |  |  |
| Heart rate | +0.012 | 0.000 |  |  |
| Previous HF hospitalization in last year | - 0.061 | 0.301 |  |  |
| Hospitalization, type of visit | +0.470 | 0.000 | +0.238 | 0.004 |
| NT-proBNP | +0.454 | 0.000 | +0.548 | 0.000 |
| Troponin T | +0.001 | 0.985 |  |  |
| Hemoglobin | - 0.043 | 0.003 | +0.079 | 0.014 |
| Sodium | - 0.053 | 0.000 | - 0.052 | 0.001 |
| Potassium | - 0.161 | 0.001 |  |  |
| eGFR | +0.494 | 0.000 |  |  |
| Albumine | - 0.022 | 0.000 |  |  |
| BUN | +0.128 | 0.005 |  |  |
| CRP | +0.219 | 0.000 |  |  |
| HDL | - 0.536 | 0.000 | - 0.255 | 0.028 |
| IL-6 | +0.364 | 0.000 |  |  |
| Leucocytes | +0.032 | 0.001 |  |  |
| ASAT | +0.409 | 0.000 |  |  |
| ALAT | +0.189 | 0.000 |  |  |
| γ-GT | +0.459 | 0.000 |  |  |
| Alkaline phosphatase | +0.488 | 0.000 |  |  |
| Atrial fibrillation | +0.155 | 0.005 |  |  |
| Diabetes mellitus | +0.097 | 0.097 |  |  |
| COPD | +0.046 | 0.526 |  |  |
| Renal disease | +0.289 | 0.000 |  |  |
| Smoking | +0.024 | 0.542 |  |  |
| Malignancy | +0.258 | 0.072 |  |  |
| Device therapy | - 0.037 | 0.112 |  |  |
| Loop diuretics | +0.367 | 0.376 |  |  |
| Beta-blockers | - 0.264 | 0.000 |  |  |
| ACE-inhibitors/ARB | - 0.156 | 0.010 |  |  |
| MRA | +0.042 | 0.437 |  |  |
| Oral anticoagulants | - 0.007 | 0.907 |  |  |
| CA15-3 |  |  |  |  |
| Age | +0.005 | 0.000 | +0.004 | 0.006 |
| Sex | +0.018 | 0.466 |  |  |
| BMI | - 0.002 | 0.341 |  |  |
| Ischemic etiology | +0.063 | 0.004 |  |  |
| NYHA class III/IV | +0.042 | 0.060 |  |  |
| LVEF | - 0.000 | 0.904 |  |  |
| Oedema | +0.026 | 0.283 |  |  |
| Systolic blood pressure | - 0.001 | 0.232 |  |  |
| Heart rate | - 0.001 | 0.038 |  |  |
| Previous HF hospitalization in last year | +0.055 | 0.017 |  |  |
| Hospitalization, type of visit | +0.000 | 0.979 |  |  |
| NT-proBNP | +0.009 | 0.559 |  |  |
| Troponin T | - 0.012 | 0.483 |  |  |
| Hemoglobin | - 0.014 | 0.014 |  |  |
| Sodium | - 0.013 | 0.000 | - 0.013 | 0.001 |
| Potassium | - 0.036 | 0.061 | - 0.082 | 0.006 |
| eGFR | - 0.000 | 0.998 |  |  |
| Albumine | - 0.000 | 0.830 |  |  |
| BUN | +0.080 | 0.000 | +0.120 | 0.000 |
| CRP | +0.028 | 0.003 |  |  |
| HDL | - 0.048 | 0.107 |  |  |
| IL-6 | +0.025 | 0.018 |  |  |
| Leucocytes | - 0.005 | 0.223 |  |  |
| ASAT | +0.034 | 0.105 |  |  |
| ALAT | - 0.030 | 0.063 |  |  |
| γ-GT | +0.009 | 0.601 |  |  |
| Alkaline phosphatase | +0.136 | 0.000 | +0.149 | 0.000 |
| Atrial fibrillation | +0.021 | 0.335 |  |  |
| Diabetes mellitus | +0.046 | 0.045 |  |  |
| COPD | - 0.011 | 0.704 |  |  |
| Renal disease | +0.117 | 0.000 |  |  |
| Smoking | - 0.003 | 0.854 |  |  |
| Malignancy | +0.067 | 0.228 |  |  |
| Device therapy | +0.020 | 0.026 |  |  |
| Loop diuretics | - 0.015 | 0.925 |  |  |
| Beta-blockers | - 0.013 | 0.648 |  |  |
| ACE-inhibitors/ARB | - 0.055 | 0.020 |  |  |
| MRA | +0.011 | 0.593 |  |  |
| Oral anticoagulants | +0.001 | 0.978 |  |  |
| CA19-9 |  |  |  |  |
| Age | +0.008 | 0.000 |  |  |
| Sex | - 0.034 | 0.565 |  |  |
| BMI | - 0.001 | 0.789 |  |  |
| Ischemic etiology | +0.084 | 0.114 |  |  |
| NYHA class III/IV | +0.177 | 0.001 | +0.253 | 0.015 |
| LVEF | +0.000 | 0.979 |  |  |
| Oedema | +0.332 | 0.000 | +0.271 | 0.006 |
| Systolic blood pressure | - 0.003 | 0.023 |  |  |
| Heart rate | +0.001 | 0.386 |  |  |
| Previous HF hospitalization in last year | +0.164 | 0.003 |  |  |
| Hospitalization, type of visit | +0.102 | 0.001 |  |  |
| NT-proBNP | +0.117 | 0.001 |  |  |
| Troponin T | +0.015 | 0.722 |  |  |
| Hemoglobin | - 0.025 | 0.076 |  |  |
| Sodium | - 0.024 | 0.000 |  |  |
| Potassium | - 0.095 | 0.042 |  |  |
| eGFR | +0.169 | 0.000 | +0.207 | 0.007 |
| Albumine | - 0.009 | 0.004 |  |  |
| BUN | +0.144 | 0.001 |  |  |
| CRP | +0.022 | 0.351 |  |  |
| HDL | - 0.020 | 0.783 |  |  |
| IL-6 | +0.133 | 0.000 |  |  |
| Leucocytes | +0.004 | 0.679 |  |  |
| ASAT | +0.233 | 0.000 | +0.274 | 0.012 |
| ALAT | +0.044 | 0.259 |  |  |
| γ-GT | +0.176 | 0.000 |  |  |
| Alkaline phosphatase | +0.215 | 0.008 |  |  |
| Atrial fibrillation | +0.212 | 0.000 |  |  |
| Diabetes mellitus | +0.211 | 0.000 |  |  |
| COPD | +0.061 | 0.376 |  |  |
| Renal disease | +0.187 | 0.001 |  |  |
| Smoking | - 0.051 | 0.179 |  |  |
| Malignancy | +0.145 | 0.289 |  |  |
| Device therapy | +0.041 | 0.069 |  |  |
| Loop diuretics | +0.624 | 0.114 |  |  |
| Beta-blockers | - 0.077 | 0.269 |  |  |
| ACE-inhibitors/ARB | - 0.081 | 0.162 |  |  |
| MRA | - 0.012 | 0.811 |  |  |
| Oral anticoagulants | +0.106 | 0.048 |  |  |
| CEA |  |  |  |  |
| Age | +0.005 | 0.000 |  |  |
| Sex | - 0.037 | 0.279 |  |  |
| BMI | - 0.008 | 0.006 | - 0.012 | 0.009 |
| Ischemic etiology | +0.083 | 0.006 |  |  |
| NYHA class III/IV | +0.081 | 0.009 |  |  |
| LVEF | +0.001 | 0.579 |  |  |
| Oedema | +0.120 | 0.000 | +0.167 | 0.004 |
| Systolic blood pressure | - 0.000 | 0.662 |  |  |
| Heart rate | +0.002 | 0.030 |  |  |
| Previous HF hospitalization in last year | +0.055 | 0.088 |  |  |
| Hospitalization, type of visit | +0.033 | 0.058 |  |  |
| NT-proBNP | +0.049 | 0.017 |  |  |
| Troponin T | +0.023 | 0.291 |  |  |
| Hemoglobin | - 0.010 | 0.201 |  |  |
| Sodium | - 0.010 | 0.012 |  |  |
| Potassium | +0.024 | 0.363 |  |  |
| eGFR | +0.043 | 0.093 |  |  |
| Albumine | - 0.003 | 0.056 |  |  |
| BUN | +0.082 | 0.001 |  |  |
| CRP | +0.041 | 0.002 |  |  |
| HDL | +0.008 | 0.849 |  |  |
| IL-6 | +0.080 | 0.000 |  |  |
| Leucocytes | +0.015 | 0.007 | +0.017 | 0.049 |
| ASAT | +0.068 | 0.022 |  |  |
| ALAT | - 0.006 | 0.799 |  |  |
| γ-GT | +0.061 | 0.010 |  |  |
| Alkaline phosphatase | +0.047 | 0.315 |  |  |
| Atrial fibrillation | +0.069 | 0.022 |  |  |
| Diabetes mellitus | +0.034 | 0.287 |  |  |
| COPD | +0.194 | 0.000 |  |  |
| Renal disease | +0.109 | 0.001 | +0.132 | 0.018 |
| Smoking | +0.155 | 0.000 | +0.127 | 0.001 |
| Malignancy | +0.022 | 0.773 |  |  |
| Device therapy | +0.015 | 0.227 |  |  |
| Loop diuretics | +0.247 | 0.277 |  |  |
| Beta-blockers | - 0.082 | 0.040 |  |  |
| ACE-inhibitors/ARB | - 0.050 | 0.129 |  |  |
| MRA | - 0.016 | 0.602 |  |  |
| Oral anticoagulants | +0.024 | 0.430 |  |  |
| CYFRA 21-1 |  |  |  |  |
| Age | +0.014 | 0.000 |  |  |
| Sex | +0.036 | 0.195 |  |  |
| BMI | - 0.005 | 0.015 | - 0.019 | 0.000 |
| Ischemic etiology | +0.175 | 0.000 |  |  |
| NYHA class III/IV | +0.103 | 0.000 |  |  |
| LVEF | +0.007 | 0.000 |  |  |
| Oedema | +0.094 | 0.001 |  |  |
| Systolic blood pressure | +0.001 | 0.208 |  |  |
| Heart rate | - 0.003 | 0.000 |  |  |
| Previous HF hospitalization in last year | +0.114 | 0.000 |  |  |
| Hospitalization, type of visit | +0.013 | 0.369 |  |  |
| NT-proBNP | +0.077 | 0.000 |  |  |
| Troponin T | +0.085 | 0.000 |  |  |
| Hemoglobin | - 0.080 | 0.000 | - 0.052 | 0.002 |
| Sodium | - 0.005 | 0.099 |  |  |
| Potassium | +0.071 | 0.001 |  |  |
| eGFR | - 0.003 | 0.877 |  |  |
| Albumine | - 0.007 | 0.000 |  |  |
| BUN | +0.208 | 0.000 | +0.164 | 0.003 |
| CRP | +0.009 | 0.414 |  |  |
| HDL | +0.006 | 0.868 |  |  |
| IL-6 | +0.071 | 0.000 |  |  |
| Leucocytes | +0.014 | 0.002 | +0.024 | 0.005 |
| ASAT | - 0.053 | 0.030 |  |  |
| ALAT | - 0.109 | 0.000 |  |  |
| γ-GT | - 0.024 | 0.231 |  |  |
| Alkaline phosphatase | +0.066 | 0.084 |  |  |
| Atrial fibrillation | +0.040 | 0.106 |  |  |
| Diabetes mellitus | +0.194 | 0.000 |  |  |
| COPD | +0.122 | 0.000 |  |  |
| Renal disease | +0.330 | 0.000 | +0.175 | 0.017 |
| Smoking | - 0.049 | 0.006 |  |  |
| Malignancy | +0.139 | 0.030 | +0.298 | 0.015 |
| Device therapy | +0.016 | 0.127 |  |  |
| Loop diuretics | +0.113 | 0.567 |  |  |
| Beta-blockers | - 0.023 | 0.482 |  |  |
| ACE-inhibitors/ARB | - 0.108 | 0.000 | - 0.143 | 0.026 |
| MRA | - 0.060 | 0.015 |  |  |
| Oral anticoagulants | - 0.015 | 0.550 |  |  |
| AFP |  |  |  |  |
| Age | - 0.006 | 0.000 |  |  |
| Sex | +0.218 | 0.000 |  |  |
| BMI | - 0.018 | 0.000 | - 0.026 | 0.001 |
| Ischemic etiology | - 0.162 | 0.000 | - 0.266 | 0.003 |
| NYHA class III/IV | - 0.093 | 0.004 |  |  |
| LVEF | - 0.005 | 0.003 |  |  |
| Oedema | - 0.037 | 0.286 |  |  |
| Systolic blood pressure | - 0.000 | 0.513 |  |  |
| Heart rate | +0.003 | 0.001 |  |  |
| Previous HF hospitalization in last year | - 0.166 | 0.000 |  |  |
| Hospitalization, type of visit | - 0.050 | 0.005 |  |  |
| NT-proBNP | +0.010 | 0.655 |  |  |
| Troponin T | - 0.059 | 0.021 |  |  |
| Hemoglobin | +0.071 | 0.000 | +0.057 | 0.016 |
| Sodium | +0.001 | 0.843 |  |  |
| Potassium | - 0.027 | 0.329 |  |  |
| eGFR | - 0.011 | 0.688 |  |  |
| Albumine | +0.006 | 0.000 |  |  |
| BUN | - 0.228 | 0.000 |  |  |
| CRP | - 0.053 | 0.000 | - 0.099 | 0.021 |
| HDL | +0.198 | 0.000 |  |  |
| IL-6 | - 0.074 | 0.000 |  |  |
| Leucocytes | - 0.004 | 0.465 |  |  |
| ASAT | +0.216 | 0.000 |  |  |
| ALAT | +0.152 | 0.000 |  |  |
| γ-GT | +0.126 | 0.000 |  |  |
| Alkaline phosphatase | +0.086 | 0.075 |  |  |
| Atrial fibrillation | - 0.031 | 0.325 |  |  |
| Diabetes mellitus | - 0.330 | 0.000 |  |  |
| COPD | - 0.098 | 0.018 |  |  |
| Renal disease | - 0.221 | 0.000 |  |  |
| Smoking | +0.009 | 0.693 |  |  |
| Malignancy | - 0.009 | 0.911 |  |  |
| Device therapy | - 0.029 | 0.028 |  |  |
| Loop diuretics | +0.207 | 0.384 |  |  |
| Beta-blockers | - 0.041 | 0.326 |  |  |
| ACE-inhibitors/ARB | +0.051 | 0.144 |  |  |
| MRA | - 0.016 | 0.614 |  |  |
| Oral anticoagulants | +0.035 | 0.276 |  |  |

ACE: angiotensin converting enzyme; ALAT: alanine transaminase; ARB: angiotensin receptor blocker; ASAT: aspartate transaminase; BMI: body mass index; BUN: blood urea nitrogen; COPD: chronic obstructive pulmonary disease; CRP: c-reactive protein; eGFR: estimated glomerular filtration rate; γ-GT: γ-glutamyl transpeptidase; HDL: High‐density lipoprotein; IL-6: Interleukin 6; LVEF: left ventricular ejection fraction; NT-proBNP: N-terminal pro-B-type natriuretic peptide; NYHA: New York Heart Association; MRA: mineralocorticoid receptor antagonist

**Table S6** Cox regression analysis for secondary outcomes

|  | Univariable | | Multivariable | | |
| --- | --- | --- | --- | --- | --- |
|  | (S)HR* (95% CI) | *P*-value | (S)HR* (95% CI) | *P*-value | Harrell’s C |
| CA125 |  |  |  |  |  |
| Composite endpoint | 1.20 (1.15 – 1.24) | <0.001 | 1.13 (1.08 – 1.17) | <0.001 | 0.69 |
| HF hospitalization | 1.13 (1.08 – 1.18) | <0.001 | 1.10 (1.05 – 1.15) | <0.001 |  |
| CV mortality | 1.22 (1.16 – 1.28) | <0.001 | 1.15 (1.08 – 1.22) | <0.001 |  |
| Non-CV mortality | 1.16 (1.06 – 1.28) | 0.002 | 1.12 (1.00 – 1.25) | 0.047 |  |
| CA15-3 |  |  |  |  |  |
| Composite endpoint | 1.25 (1.13 – 1.38) | <0.001 | 1.12 (1.02 – 1.24) | 0.023 | 0.69 |
| HF hospitalization | 1.17 (1.03 – 1.34) | 0.015 | 1.07 (0.94 – 1.21) | 0.339 |  |
| CV mortality | 1.30 (1.11 – 1.52) | 0.001 | 1.13 (0.96 – 1.33) | 0.140 |  |
| Non-CV mortality | 1.32 (1.00 – 1.75) | 0.055 | 1.19 (0.89 – 1.58) | 0.247 |  |
| CA19-9 |  |  |  |  |  |
| Composite endpoint | 1.12 (1.07 – 1.16) | <0.001 | 1.05 (1.01 – 1.09) | 0.023 | 0.69 |
| HF hospitalization | 1.07 (1.01 – 1.13) | 0.019 | 1.02 (0.96 – 1.07) | 0.551 |  |
| CV mortality | 1.16 (1.08 – 1.26) | <0.001 | 1.08 (1.01 – 1.16) | 0.024 |  |
| Non-CV mortality | 1.35 (1.18 – 1.54) | <0.001 | 1.28 (1.12 – 1.46) | <0.001 |  |
| CYFRA 21-1 |  |  |  |  |  |
| Composite endpoint | 1.64 (1.51 – 1.77) | <0.001 | 1.36 (1.25 – 1.48) | <0.001 | 0.69 |
| HF hospitalization | 1.29 (1.17 – 1.42) | <0.001 | 1.08 (0.97 – 1.20) | 0.155 |  |
| CV mortality | 1.62 (1.42 – 1.84) | <0.001 | 1.26 (1.09 – 1.46) | 0.002 |  |
| Non-CV mortality | 1.72 (1.43 – 2.07) | <0.001 | 1.48 (1.23 – 1.79) | <0.001 |  |
| CEA |  |  |  |  |  |
| Composite endpoint | 1.25 (1.17 – 1.34) | <0.001 | 1.15 (1.07 – 1.23) | <0.001 | 0.69 |
| HF hospitalization | 1.12 (1.03 – 1.22) | 0.012 | 1.05 (0.96 – 1.15) | 0.263 |  |
| CV mortality | 1.30 (1.17 – 1.45) | <0.001 | 1.18 (1.06 – 1.32) | 0.003 |  |
| Non-CV mortality | 1.22 (1.00 – 1.49) | 0.051 | 1.12 (0.93 – 1.37) | 0.222 |  |
| AFP |  |  |  |  |  |
| Composite endpoint | 0.87 (0.82 – 0.93) | <0.001 | 0.96 (0.89 – 1.02) | 0.206 | 0.69 |
| HF hospitalization | 0.90 (0.83 – 0.97) | 0.010 | 0.97 (0.90 – 1.05) | 0.492 |  |
| CV mortality | 0.88 (0.79 – 0.97) | 0.010 | 0.96 (0.88 – 1.07) | 0.538 |  |
| Non-CV mortality | 1.01 (0.81 – 1.28) | 0.902 | 1.16 (0.93 – 1.43) | 0.185 |  |

* Hazard ratio (HR) for the composite endpoint of all-cause mortality and HF hospitalization, subhazard ratio (SHR) for HF hospitalization, CV mortality and non-CV mortality.

Multivariable models corrected as follows:

- Composite endpoint: age, previous hospitalization, presence of oedema, NT-proBNP, systolic blood pressure at baseline, hemoglobin, HDL levels, sodium, beta-blocker
- HF hospitalization: age, previous HF hospitalization, presence of edema at baseline, systolic blood pressure at baseline, and eGFR
- CV mortality: age, BUN, NT-proBNP, troponin T, and sodium
- Non-CV mortality: age, hemoglobin, CRP, and history of malignancy
